# Theoretical Exploration of Properties of Iron–Silicon Interface Constructed by Depositing Fe on Si(111)-(7×7)

**DOI:** 10.3390/molecules28207181

**Published:** 2023-10-19

**Authors:** Jun-Qing Yin, Yan-Ping Zhang, Yong You, Zhen-Hua Wang, Jian-Qiang Zhao, Qing Peng

**Affiliations:** 1 Institute of Advanced Study, Chengdu University, Chengdu 610106, China; youyong@cdu.edu.cn (Y.Y.); wangzhenhua@cdu.edu.cn (Z.-H.W.); zhaojianqiang@cdu.edu.cn (J.-Q.Z.); 2 School of Pharmacy, Chengdu University, Chengdu 610106, China; zhangyanping@cdu.edu.cn; 3 State Key Laboratory of Nonlinear Mechanics, Institute of Mechanics, Chinese Academy of Sciences, Beijing 100190, China; pengqing@imech.ac.cn

**Keywords:** iron, Si(111)-(7×7), iron–silicon interface, CO adsorption, density functional theory

## Abstract

Exploring the properties of magnetic metal on the semiconductor surface is of great significance for the application of magnetic recording materials. Herein, DFT calculations are carried out to explore the properties of the iron–silicon interface structures (*n*Fe/DASF) formed by depositing *n* Fe atoms on the reconstructed Si(111)-(7×7) surface (DASF). The stable *n*Fe/DASF structures are studied in the cases of the adsorption and permeation of Fe atoms on the DASF. In both cases, Fe atoms are not very dispersed and prefer binding with Si atoms rather than the adsorbed Fe atoms, because the Fe-Si interaction is stronger than the Fe-Fe interaction. As the *n* value increases, the average binding energy (*E*_b_ave_) of Fe generally firstly becomes more negative and then becomes less negative, with the presence of a 7Fe wheel as a stable geometry on the upmost surface. The presence of the 7Fe wheel is attributed to the enhanced Fe-Si interaction in this wheel compared to other geometries. CO adsorption occurs at the central Fe site of the 7Fe wheel which is greatly influenced by the surrounding Si atoms but is little influenced by the additional Fe atoms in the interlayer.

## 1. Introduction

Metal–semiconductor interfaces, formed by the deposition of metallic atoms or nanoparticles on the semiconductor surface, have attracted much attention due to their practical importance in modern integrated circuit devices. Silicon is an important base material for metal–semiconductor interfaces [1,2] and is widely used in the fabrication of microelectronics, benefiting from the great improvement in the mechanical properties of silicon material at the surface contact scale from the implantation of silicon with different ions [3,4,5,6]. Based on well-developed Si-based technology, magnetic materials prepared by depositing magnetic metal on silicon [7,8] are also finding practical applications in the field of magnetic recording. The properties of magnetic recording materials depend on achieving controllable growth of individual magnetic domain clusters on the silicon [9,10,11,12]. Therefore, it is of significance to study the properties of the metal–semiconductor interfaces in the initial growth of magnetic clusters, but studies on this are lacking.

Iron is the most common 3*d* ferromagnetic metal and exhibits high spin polarization. Many studies have focused on the interface structure formed during the process of Fe deposition on the silicon substrate and have demonstrated that the mechanical properties of silicon-based iron magnetic materials can be improved by controlling the nature of the Fe/Si(111) interface structure [13,14,15]. In order to control the quality of the iron–silicon interface, it is necessary to properly control the initial process of Fe deposition. Miranda et al. [16] studied the initial stages of Fe growth on Si(111)-(7×7) and concluded that an amorphous layer with composition and density of states close to those of FeSi was formed, in agreement with reports of FeSi formation [17]. Eguchi et al. [18] performed an atomic-scale investigation of the initial stage of growth and interface formation of Fe on a H-terminated Si(111)-(1×1) surface and found the presence of a (111)-oriented body-centered-cubic (bcc) Fe cluster structure on the substrate surface. It should be noted that growth of the pure Fe or the amorphous layer required a sufficient thickness deposited, making it difficult to obtain a clear structure of the Fe/Si(111) interface at the initial stage of Fe growth. Thibaudau et al. [19] investigated the interaction of iron pentacarbonyl (Fe(CO)_5_) and ferrocene (Fe(C_5_H_5_)_2_) with Si(111)-(7×7), and found that exposure to Fe(CO)_5_ leads to the growth of much higher quality iron silicide by controlling the dissociative adsorption of Fe(CO)_5_ in which the CO easily desorbs leaving only the iron atom on the surface after a complete dissociation of the molecule, compared to the case of exposure to Fe(C_5_H_5_)_2_ where silicide carbide is formed. There are also many reports on the interaction between the gaseous molecule and Si(111)-(7×7) without a metal element [20,21,22]. Here, we have some questions as follows: (a) Are the deposited Fe atoms atomically dispersed or clustered on the silicon substrate? (b) What is the most stable structure of *n*Fe/Si(111)-(7×7) (where *n* is the number of Fe atoms on Si(111)-(7×7)) at the initial stages of Fe deposition on Si(111)-(7×7)? (c) How do the electronic properties of *n*Fe/Si(111)-(7×7) change as the number of deposited Fe atoms increases? (d) What are the reactivities of the Fe atoms on *n*Fe/Si(111)-(7×7)? Such knowledge is a basis for a deeper understanding of the properties of the iron–silicon interface, but remains unclear.

To answer these open questions, in this work we theoretically investigated the Fe-coverage-dependent properties of an iron–silicon interface structure in the early stages of Fe growth on Si(111)-(7×7). The well-reconstructed Si(111)-(7×7) substrate is a dimer-adatom-stacking-fault (DASF) model. The structures of *n*Fe/DASF are constructed by depositing Fe atoms one by one onto the DASF substrate, to clarify whether the deposited Fe atoms are atomically dispersed or clustered on the silicon. The permeation of the Fe atom from the top surface to the second and third layers is explored, and, accordingly, we placed each Fe atom on the different layers to find the most stable structures of *n*Fe/DASF at different *n* values. We then obtained the electronic properties and reactivities of the *n*Fe/DASF models and explored the dependence of these properties on the Fe content. The theoretical knowledge provides us with a good understanding of the iron–silicon interface.

## 2. Results and Discussion

### 2.1. The DASF Surface Structure

**Scheme 1:**
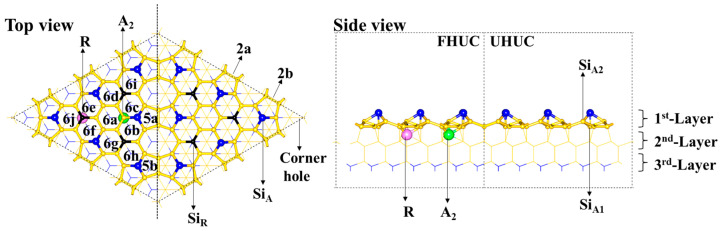
Top and side views of the DASF model and the adsorption sites for Fe in the model. The symbols Si_A_ and Si_R_ represent the Si adatom (blue spheres) and the Si rest atom (black spheres), respectively. The blue lines are for the Si atoms at the bottom of the model and the other Si atoms use the yellow spheres/lines. Each Si_A_ atom binds with one Si_A1_ and three Si_A2_ atoms. In the first layer, the possible adsorption sites are denoted by the symbols **6a**~**6j** (six-membered Si rings) and **5a**~**5b** (five-membered Si rings). The interlayer position just below the Si_R_ atom is denoted by the symbol **R** and the position just below the Si_A2_ atom is denoted by the symbol **A2**.

The DASF model consists of a faulted half-unit cell (FHUC) and an unfaulted half-unit cell (UHUC), as is shown in Scheme 1. Within each unit cell, there are three rest atoms (represented by Si_R_) and six adatoms (represented by Si_A_). The Si_R_ atoms are 0.81 Å lower than the Si_A_ atoms. The Si_R_ and Si_A_ atoms differ in their coordination environment. The coordination number of the Si_R_ atom is three and the average Si–Si bond length is 2.41 Å. The coordination number of the Si_A_ atom is four (coordinated with one Si_A1_ atom and three Si_A2_ atoms) and the average Si–Si bond length is 2.48 Å. Both the Si_A1_ and Si_A2_ atoms are four-coordinated but differ in the coordination environment, which is close to a tetrahedron for Si_A1_ but greatly distorted from a tetrahedron for Si_A2_. The Si_A_ adatom is nonequivalent sp^3^ hybridized and strongly polarized. On the DASF surface, there are two kinds of five-membered rings (represented by **5a** and **5b**, respectively) and three kinds of six-membered Si rings (represented by **6a** for the first kind, **6b**~**6g** for the second kind, and **6h**~**6j** for the third kind, respectively), according to symmetry. The main structural difference between the two cells is that the third layer Si atoms (blue lines) are located just below the center of these six-membered Si rings in the FHUC, whereas the second layer Si atoms (yellow lines) are located just below the center of these six-membered Si rings in the UHUC. In addition, there is a hole in the corner of the cell. Along the contacted edge of the two half-unit cells are dimers consisting of two Si atoms on the surface, which are represented by **2a** and **2b** with lengths of 2.43 Å and 2.46 Å, respectively.

### 2.2. The Adsorption of Fe Atoms on the Upmost Surface

**Scheme 2:**
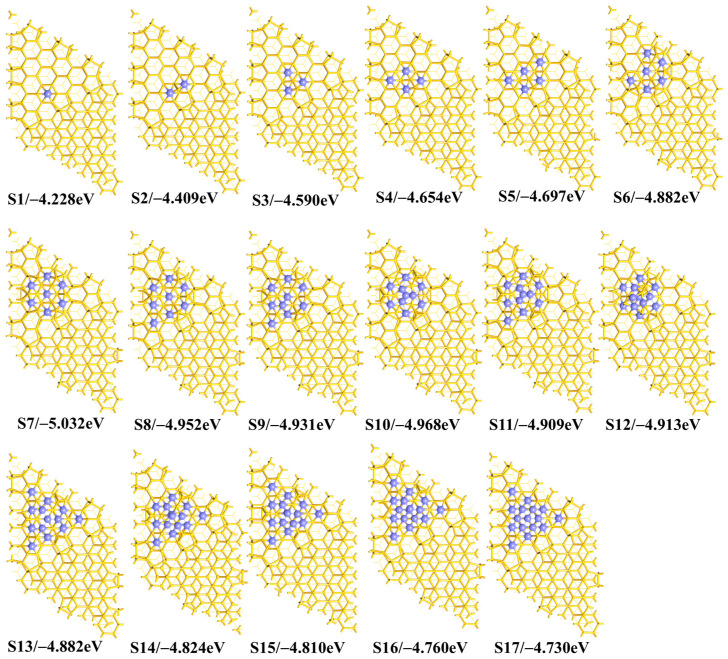
Geometries with the averaged binding energy (*E*_b_ave_) of the adsorbed Fe atoms on the FHUC. The blue color indicates Fe and the yellow color indicates Si.

For a single Fe atom in the FHUC region, the binding energy (*E*_b_) is −4.05 eV at the **6a** site, −4.23 eV at the **6b** site, and −3.99 eV at the **5a**, **5b**, and **6h** sites. It indicates that the Fe atom is adsorbed at the **6b** site, as shown by the **S1** model in Scheme 2. In the **S1** model, the distance between the Fe and Si_A_ atoms is 0.12 Å shorter than that between the Fe and Si_R_ atoms (2.28 Å vs. 2.40 Å). Similarly, in the UHUC region, the Fe atom is more stable at the **6b** site than at any other site; the distance between the Fe and Si_A_ atoms is closer to that between the Fe and Si_R_ atoms (2.33 Å and 2.30 Å, respectively). However, the *E*_b_ value for the Fe atom at the **6b** site in the FHUC region is more negative by 0.25 eV than that in the UHUC region (−4.23 eV vs. −3.98 eV). The result shows that the Fe atom prefers the **6b** site in the FHUC region rather than that in the UHUC region, consistent with the experimental result by Thibaudau et al. [19] that the dissociated Fe(CO)_5_ leaves the Fe atom in the FHUC region where the Si_R_ atoms are the intrinsic sites for dissociative adsorption of Fe(CO)_5_. Next, we focused on the adsorption of more Fe atoms in the FHUC region.

By keeping the first Fe atom at the **6b** site and moving the second Fe atom from one site to another, we studied the adsorption of the second Fe atom and accessed the system stability according to the *E*_b_ave_ value. The *E*_b_ave_ value is −4.25 eV at the **6a** site, −4.41 eV at the **6c** site, −4.20 eV at the **6d** site, −4.08 eV at the **6e** site, −4.08 eV at the **6f** site, −4.22 eV at the **6g** site, −4.24 eV at the **6h** site, −4.18 eV at the **6i** site, −4.07 eV at the **6j** site, and −4.40 eV at the **5a** site for the second Fe atom. We also explored the adsorption positions around or above the first Fe atom to check the possibility of the Fe–Fe bond formation and found that the *E*_b_ave_ values range from −3.75 eV to −4.03 eV. The result indicates that the second Fe atom prefers the **6c** site (Scheme 2 **S2**); the Fe-Si interaction is stronger than the Fe-Fe interaction because there is no Fe–Fe bond formed. Because the difference in the *E*_b_ave_ values between the cases with and without the Fe–Fe bond formation is large, we will not discuss the case of Fe adsorption with the Fe–Fe bond formation when the number of Fe atoms is not very large.

Similarly, the geometry for the coadsorption of three Fe atoms is optimized by keeping two Fe atoms at the **6b** and **6c** sites, and testing the adsorption of the third Fe atom at each six-membered Si ring. In this case, the coadsorption of three Fe atoms is the most stable with the *E*_b_ave_ value of −4.59 eV, where they form a minimum triangular pattern by being distributed, respectively, at the **6b**, **6c**, and **6a** sites, as shown by the **S3** model in Scheme 2. In the **S3** model, the distance between the Fe atoms at the **6b** and **6c** sites is 3.694 Å, and that between the Fe atoms at the **6a** and **6b** (or **6c**) sites is greater at 4.022 Å. Also, we expanded the Fe positions to those five-membered Si rings. It may be possible to find a stable coadsorption of the three Fe atoms by placing two of them, respectively, at the **6b** and **5a** sites, since the *E*_b_ave_ for only two Fe atoms adsorbed at the **6b** and **5a** sites is just 0.01 eV higher than the most stable adsorption at the **6b** and **6c** sites. In this case, however, we found that the *E*_b_ave_ value for three Fe atoms, respectively, at the **6b**, **5a**, and **6c** sites becomes less negative by 0.02 eV than that for three Fe atoms, respectively, at the **6b**, **6c**, and **6a** sites. Thus, three Fe atoms adsorbed, respectively, at the **6b**, **6c**, and **6a** sites lead to the **S3** model as the most stable geometry.

**Figure 1:**
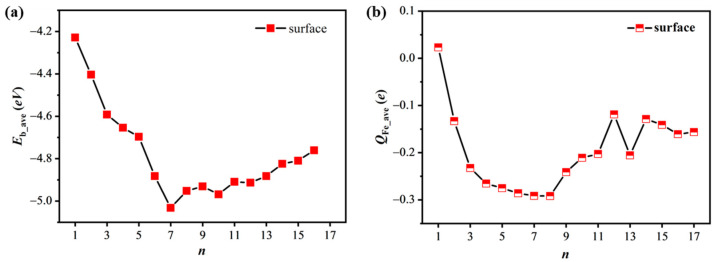
Variations of the averaged binding energy (*E*_b_ave_, eV) (**a**) and the averaged Bader charge (*Q*_Fe_ave_, *e*) of Fe atoms (**b**) against the number *n* for Fe atoms in the case that Fe atoms are on the surface of FHUC.

As the number *n* is further increased, the *E*_b_ave_ value becomes more negative until it reaches the minimum value of −5.032 eV when there are seven Fe atoms on the surface (Scheme 2 **S7**), as shown in Figure 1a and Table S1 of the Supplementary Materials. The fourth and fifth Fe atoms are adsorbed at the **6d** and **6g** sites, respectively. The sixth and seventh Fe atoms are adsorbed at the **6e** and **6s** sites, respectively. We concluded that the Fe atoms prefer to stay at the six-membered ring sites close to each other on the surface. In this way, the seven Fe atoms form a wheel-like 7Fe geometry, with one Fe atom at the center and the other six atoms around the center. The presence of the wheel-like 7Fe geometry is related to the location of the first free Fe atoms, according to the energetically preferred path of these Fe atoms. Since the triangular pattern makes the system stable, as demonstrated theoretically by the case of coadsorption of three Fe atoms in the early part of this work, more triangular structures are generated by these seven Fe atoms through forming the wheel-like 7Fe geometry.

Compared to the *n* = 7 case, the *E*_b_ave_ value becomes less negative as the number *n* increases. The eighth Fe atom is just beside the wheel (Scheme 2 **S8**) but the ninth Fe atom is above the wheel (Scheme 2 **S9**). When there are ten Fe atoms on the surface, the last three Fe atoms are all above the wheel (Scheme 2 **S10**). As the number *n* increases from 8 to 13, the *E*_b_ave_ value presents a slight oscillation. In the *n* = 13 case, all the six-membered ring sites are covered by Fe atoms, as is shown by Scheme 2 **S13**. As the number *n* increases from 13 to 17, the *E*_b_ave_ value becomes less negative, because of the formation of fewer Fe–Si bonds but more Fe–Fe bonds. A general trend of the *E*_b_ave_ ~ *n* variation thus is clear, that the *E*_b_ave_ value first increases and then decreases, and reaches the minimum value at *n* = 7, from which we conclude that the S7 model is more stable than the others in the case of Fe adsorption only on the surface.

Here, we wish to discuss the reason(s) for the trend of the *E*_b_ave_ ~ *n* variation. In the case of a single Fe atom (Scheme 2 **S1**), the Fe atom is positively charged by 0.023 *e*, which is consistent with common sense in that the Fe atom has slightly lower electronegativity than the silicon atom. In the cases of *n* > 1, however, these *n*Fe atoms are negatively charged in the most stable geometries, because there are some Si atoms shared by these Fe atoms which limits the charge transfer from Fe to Si but facilitates the reversed charge transfer. The result is in agreement with the experimental result [17] that the electronic binding energy of the Fe becomes smaller after the deposition of Fe on DASF. As shown in Figure 1b, the average Bader charge (*Q*_Fe_ave_) of Fe atoms becomes more negative from the cases *n* = 1 (Scheme 2 **S1**) to *n* = 7 (Scheme 2 **S7**) and then less negative as the number *n* increases to 12. This trend of the *Q*_Fe_ave_ ~ *n* variation is very similar to that of the *E*_b_ave_ ~ *n* variation. The charge transfer is strongest at *n* = 7. In the **S7** case, the Bader charge of the central Fe atom is −0.387 *e*, which is much more negative than the *Q*_Fe_ave_ value of −0.276 *e* for the other six Fe atoms, because the central Fe atom is surrounded by fewer second-order neighboring Si atoms. However, the further increased Fe atoms interact weakly with the model. Note that the *Q*_Fe_ave_ value in the case of *n* = 12 (Scheme 2 **S12**) is less negative than in the cases of *n* = 11 and *n* = 13 (Scheme 2 **S11** and **S13**), because there are more Fe–Fe bonds presented at *n* = 12. These results suggest that what makes the wheel-like 7Fe geometry relatively stable is structurally due to the greater number of triangular structures formed by the Fe atoms than in the cases of *n* < 7 and electronically due to the stronger charge transfer from Si to Fe atoms than the cases of *n* > 7.

### 2.3. The Determining Factor(s) for Stabilizing the Wheel-like 7Fe Geometry

As is discussed above, the wheel-like 7Fe geometry has more triangular structures than other geometries generated by these seven Fe atoms. Next, it is important to study the stability of the minimum triangular structure at *n* = 3 (Scheme 2 **S3**) to understand the determining factor(s) for stabilizing the wheel-like 7Fe geometry.

**Scheme 3:**
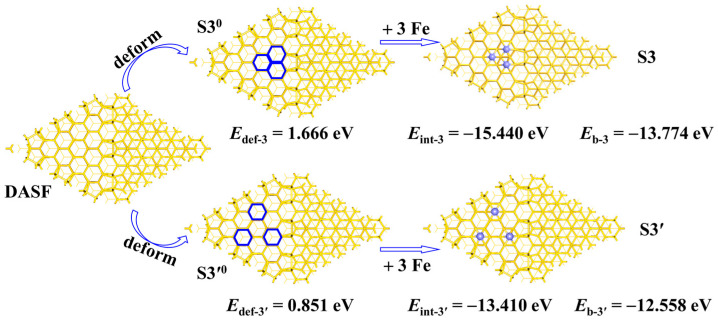
Energy changes along the assumed procedure to form the different structures of 3Fe/DASF (**S3** and **S3′**) from DASF and 3Fe atoms. The positions of all the atoms in **S3^0^** and **S3′^0^** are taken to be the same as those in **S3** and **S3′**, respectively. The blue balls indicate the Fe atoms, the blue hexagons show the deformed adsorption site, and the yellow color indicates Si.

In Scheme 3, we assumed a procedure to generate the **S3** structure and compared it with the formation process of the larger triangular structure **S3′**. The **S3** structure is 1.216 eV more stable than the **S3′** structure, as indicated by the difference between the *E*_b-3_ and *E*_b-3′_ values. Similar methods are often used to investigate the interaction between two moieties in a system [23,24]. In the procedure to generate the **S3** structure, the DASF surface is first distorted to the structure **S3^0^** taken to be same as that in **S3**; the destabilization energy *E*_def-3_ in this step is defined by the equation *E*_def-3_ = *E*_S3_^0^ − *E*_DASF_, where the subscript ‘‘def’’ means that the DASF geometry is deformed like that in the **S3^0^** model; *E*_S3_^0^ is the total energy of the structure **S3^0^**; and *E*_DASF_ is the total energy of the DASF substrate. Lastly, three Fe atoms are added to the **S3^0^** structure, affording the **S3** structure; in this step, the stabilization energy *E*_int-3_ is defined by the equation *E*_int-3_ = *E*_S3_ − (*E*_S3_^0^ + 3 *E*_Fe_), where *E*_Fe_ is the total energy of the Fe atom. Obviously, the sum of *E*_def-3_ and *E*_int-3_ is equal to the value of the *E*_b-3_ value. Similarly, for the procedure to generate the **S3** structure, the DASF surface is first distorted to the structure **S3′^0^** taken to be same as that in **S3′**. The destabilization energy *E*_def-3_**_′_** in this step is defined by the equation *E*_def-3_**_′_** = *E*_S3_**_′_**^0^ − *E*_DASF_, where the subscript ‘‘def’’ means that the DASF geometry is deformed like that in the **S3′^0^** model; *E*_S3_**_′_**^0^ is the total energy of the structure **S3′^0^**. Finally, three Fe atoms are added to the **S3′^0^** structure, with the formation of the **S3′** structure; in this step, the stabilization energy *E*_int-3_**_′_** is defined by the equation *E*_int-3′_ = *E*_S3_**_′_** − (*E*_S3_**_′_**^0^ + 3 *E*_Fe_). The sum of *E*_def-3_**_′_** and *E*_int-3_**_′_** is equal to the value of the *E*_b-3_**_′_** value.

The result shows that the *E*_int-3_ value in the **S3** case is considerably more negative by 2.030 eV than the *E*_int-3′_ value in the **S3′** case (−15.440 eV vs. −13.410 eV), meaning that the Fe-Si interaction is stronger in the **S3** geometry than in the **S3′** geometry. The *Q*_Fe_ave_ value of the 3Fe atoms is −0.232 *e* in the **S3** geometry but 0.024 *e* in the **S3′** geometry, indicating that the charge transfer between the Fe and Si atoms is stronger in the **S3** geometry than in the **S3′** geometry, supporting the change in the *E*_int_ values. The *E*_def-3_ value is much more positive by 0.81 eV than the *E*_def-3′_ value (1.661 eV eV vs. 0.851 eV). This is reasonable because the large deformation is usually caused by the strong interaction. From the **S3** case to the **S3′** case, the decreased *E*_def_ value (0.81 eV) from the **S3^0^** geometry to the **S3′^0^** geometry is much smaller than the increased *E*_int_ value (2.03 eV), showing that the *E*_int_ term plays a more important role in stabilizing the **S3** geometry than does the **S3′** geometry. It is indirectly proved that the system would become stable when the Fe atoms are clustered but without the presence of Fe–Fe bonds when the number of Fe atoms is not too large.

Therefore, the presence of a 7Fe wheel in the adsorption of Fe atoms is attributed to the enhanced Fe-Si interaction compared to the other geometries with the Fe atoms more dispersed.

### 2.4. The Permeation of Fe Atoms into the Interlayer

**Figure 2:**
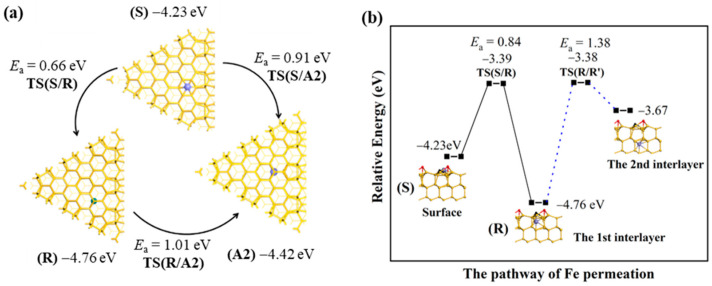
(**a**) The transition **S→R** of an Fe atom from the surface to position **R** just below Si_R_ through the transition state **TS(S/R)**, the transition **S→A2** of an Fe atom from the surface to position **A2** just below Si_A2_ through **TS(S/A2)**, and the transition **R→A2** of an Fe atom from position **R** to position **A2** through **TS(R/A2)**. (**b**) Energetic profiles of Fe atoms permeating from the surface to the first and second interlayers.

The thermodynamic stability of Fe located in the interlayer is first compared. Below the Si_R_ and Si_A2_ atoms are large enough spaces for Fe exitance, but with the binding energy of −4.76 eV and −4.42 eV, respectively. The difference between these two values is mainly resulting from the difference in the coordination environment that the Si_R_ atom is three-coordinated while the Si_A2_ atom is four-coordinated. Thus, the Fe atom is energetically more stable just below the Si_R_ atom than below the Si_A2_ atom. Then, the kinetic stability of Fe located in the interlayer is considered, as is shown in Figure 2a. The activation barrier (*E*_a_) of the **S**→**R** step is 0.66 eV for the Fe atom on the surface permeating to position **R** just below the Si_R_ atom through the transition state **TS(S/R)**, which is smaller than the 0.91 eV of the **S**→**A2** step for the Fe atom on the surface permeating to position **A2** just below the Si_A2_ atom through **TS(S/A2)**, indicating that the surface Fe atom shifts to position **R** more easily than to position **A2**. The *E*_a_ value of the **R**→**A2** step is 1.01 eV for the Fe atom just below the Si_R_ atom moving to position **A2** through **TS(R/A2)**; this step is endothermic by 0.32 eV. Since the total energy of **TS(R/A2)** is 0.43 eV lower than that of **TS(S/A2)**, the surface Fe kinetically prefers to move first to position **R** and then to position **A2** (**S**→**R**→**A2**) rather than directly to position **A2** (**R**→**A2**). Thermodynamically, the Fe atom at position **R** is more stable than at position **A2**. As is shown in Figure 2b, further permeation into the deep layer is difficult due to the large *E*_a_ value of 1.38 eV and endothermicity of 1.09 eV, so the thickness for Fe deposition is about 0.6 nm. These results suggested that the Fe atom at position **R** is thermodynamically and kinetically stable. Next, we expanded the discussion to the location of Fe atoms permeating freely into the first interlayer.

**Scheme 4:**
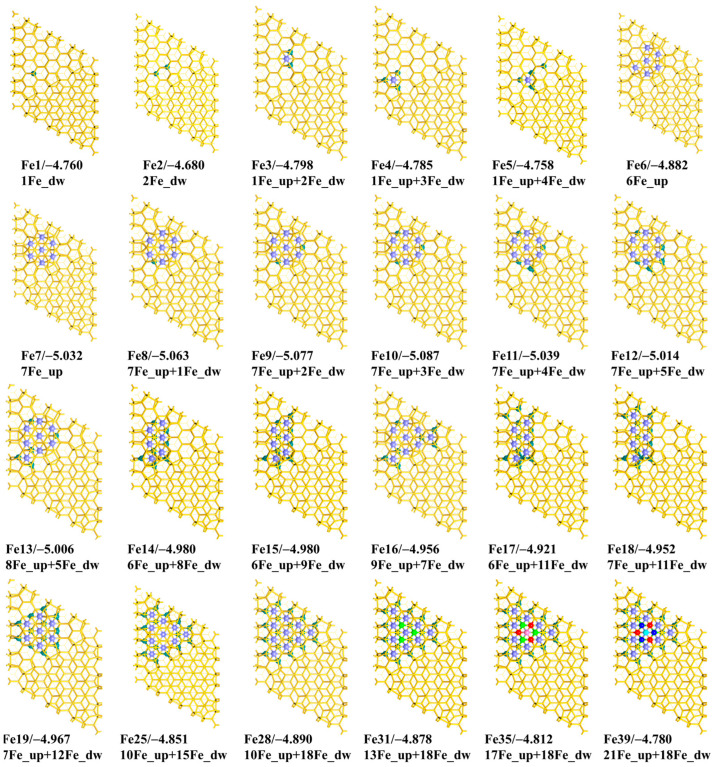
Geometries with averaged binding energy (*E*_b_ave_, eV) of Fe deposited on the FHUC in the case that the Fe atom permeates freely into the first interlayer (Fe-dw: dark green). The Fe atoms in the different layers are labeled in different colors, shown by Fe-up1 in purple, Fe-up2 in green, Fe-up3 in red, Fe-up4 in pink, Fe-up5 in blue, and Fe-up6 in cyan.

**Figure 3:**
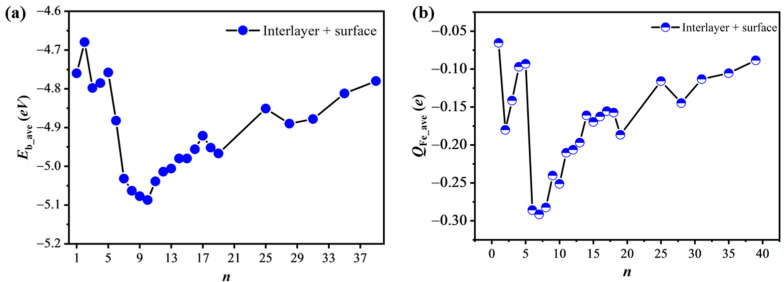
Variation of the averaged binding energy (*E*_b_ave_, *eV*) (**a**) and the averaged Bader charge (*Q*_Fe_ave_, *e*) of Fe atoms (**b**) against the number *n* for Fe atoms in the case that Fe atoms freely permeate from the surface to the first interlayer in the FHUC area.

As is shown in Scheme 4, the most stable structures of *n*Fe deposition are found with *x* Fe atoms deposited to the upmost surface (represented by *x*Fe_up) and *y* Fe atoms permeating into the first interlayer (represented by *y*Fe_dw) (*n* = *x* + *y*). When *n* = 2, the most stable geometry has one Fe atom at position **R** and the other Fe atom at position **A2** (Scheme 4 **Fe1** and **Fe2**). Because a single Fe atom is the most stable at one of the R positions, we thus checked the other **R** positions for the second Fe atom when the first Fe atom does not move but found that the *E*_b_ave_ value in this case will decrease slightly (by 0.01 eV). When *n* = 3, 4, and 5 (Scheme 4 **Fe3**, **Fe4**, and **Fe5**, respectively), there is only one Fe atom adsorbed on the six-membered ring. When *n* = 6 and 7 (Scheme 4 **Fe6** and **Fe7**), all the Fe atoms are adsorbed on the surface. In particular, the most stable geometry at *n* = 7 (Scheme 4 **Fe7**) is the same as the wheel 7Fe geometry above (Scheme 2 **S7**), even when the Fe atoms are considered to penetrate freely from the surface into the interlayer. In the cases from *n* = 7 to *n* = 13, the most stable geometry still appears to be the wheel 7Fe structure. As shown in Figure 3a and Table S2 of the Supplementary Materials, the *E*_b_ave_ value first becomes more negative and then less negative, and reaches the minimum −5.09 eV at *n* = 10 (Scheme 4 **Fe10**). Until the number *n* increases to 28 (Scheme 4 **Fe28**), all the positions in the interlayer are occupied by 18 Fe atoms and all the positions on the six-membered rings are adsorbed by the remaining 10 Fe atoms. To further increase the *n* value, the *E*_b_ave_ value become much smaller than at *n* = 28, due to the formation of Fe–Fe bonds, as shown by the **Fe31**, **Fe35**, and **Fe39** models in Scheme 4. These results show that the **Fe10** model is more stable than the others in the case that the Fe atom permeates freely from the surface to the interlayer.

Although we have obtained the geometries of *n*Fe/DASF with the most negative *E*_b_ave_ values, there are limitations in a real experiment at finite temperatures. The influence of the entropy effect and phononic contributions to the free energy, which we have not considered in this work, might change the relative stabilities of geometries with and without Fe permeation, because the differences in *E*_b_ave_ values between two models are not very large. For example, the difference in the *E*_b_ave_ values between the **Fe7** and **Fe10** models is just 0.055 eV. When considering such an influence, these energy differences will become smaller and the relative stability of them might be altered. Therefore, it is difficult to conclude which phase(s) would occur or coexist in a real situation at finite temperatures, and it is not clear whether Fe diffuses into the surface or not with the present approach.

In the case of Fe permeation, the *E*_b_ave_ ~ *n* variation is generally similar to the trend of the *Q*_Fe_ave_ ~ *n* variation (Figure 3b), suggesting that the bonding interaction is the main determining factor for the trends. The *Q*_Fe_ave_ value of two Fe atoms (Scheme 4 **Fe2**) is −0.18 *e*, which is 0.11 *e* more negative than the −0.07 *e* for the single Fe atom (Scheme 4 **Fe1**). The spin densities of the two Fe atoms are 1.58 *μ*_B_ and −0.69 *μ*_B_, which are much smaller than the 1.74 *μ*_B_ for the single Fe atom, suggesting that the spin-pairing interaction between the Fe and Si atoms is stronger in **Fe2** than in **Fe1**. From *n* = 3 to *n* = 17, the *Q*_Fe_ave_ value first becomes more negative and then less negative with the minimum value presented at *n* = 7, as shown in Figure 3b. Although the *E*_b_ave_ value at *n* = 10 (Scheme 4 **Fe10**) is 0.055 eV more negative than that at *n* = 7 (Scheme 4 **Fe7**), the *Q*_Fe_ave_ value is 0.04 *e* less negative at *n* = 10 than that at *n* = 7. The average spin density at *n* = 10 is 0.06 *μ*_B_, which is smaller than the 0.15 *μ*_B_ at *n* = 7, indicating that the spin-pairing interaction between the Fe atoms and Si atoms is stronger at *n* =10 than at *n* = 7. In the **Fe10** model, the Bader charge of the central Fe of the “7Fe wheel” is −0.348 *e*; the *Q*_Fe_ave_ value of the six Fe atoms along the ring of the “7Fe wheel” is −0.288 *e* and that of the three Fe atoms below the “7Fe wheel” is −0.145 *e*. In the cases from the **S7** model (Scheme 2) to the **Fe10** model (Scheme 4), the Bader charge of the central Fe of the “7Fe wheel” becomes less negative by 0.039 *e* while the *Q*_Fe_ave_ value for the six Fe atoms along the ring of the “7Fe wheel” becomes more negative by 0.012 *e*. From these results, it can be predicted that the reactivities of the two models are similar, as we will discuss below, because these differences are not very large.

### 2.5. CO Adsorption

**Scheme 5:**
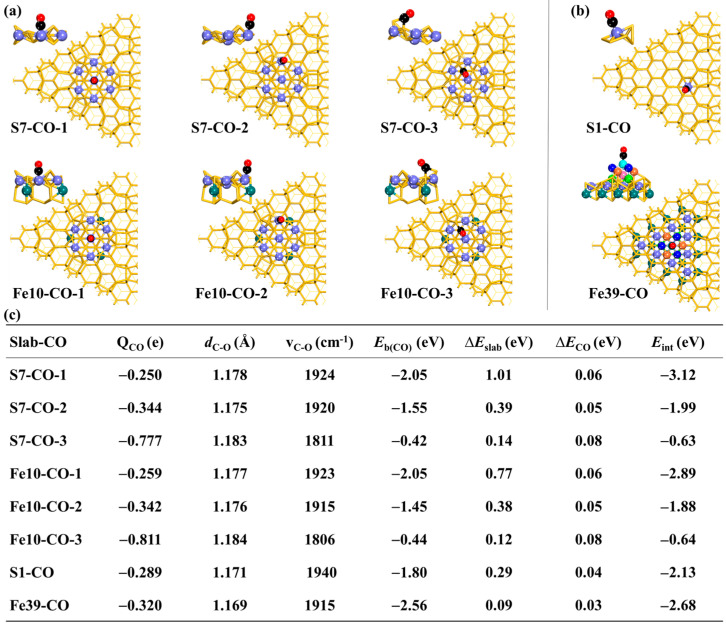
Geometries and energies of the adsorptions of one CO molecule on the slab models of nFe/DASF. The geometries of the CO adsorbed on the models of **S7** and **Fe10** (**a**), and on the models of **S1** and **Fe39** (**b**). The corresponding binding energy (*E*_b_, eV), Bader change (*Q*, *e*), C−O distance (*d*_C-O_, Å), and C−O stretching frequencies (*v*_CO_, cm^−1^), as well as the deformation energies (∆*E*_slab_ and ∆*E*_CO_) of the slab models and CO molecule, and the interaction energies (*E*_int_) between the slab model and CO molecule (**c**). All the energies are in eV. The C and O atoms of CO are in black and red colors, respectively.

The surface reactivity of *n*Fe/DASF is explored taking CO adsorption to them as an example, because CO adsorption is often studied to evaluate the surface reactivity of many materials. Koo et al. [25] carried out experiments and theoretical calculations about CO adsorption on the DASF without Fe atoms, and reported that the adsorption of CO molecules occurs on the Si_A_ atoms. However, the interaction of CO with the Fe atoms on the DASF surface remains unclear. We studied the adsorption of CO on the models of **S7** in Scheme 2 and **Fe10** in Scheme 4, because they are more stable than other geometries. As shown in Scheme 5, the geometries and energies of the CO adsorption on the **S7** and **Fe10** models are parallel to those of the CO adsorption on the **S1** and **Fe39** models for easy comparison. Generally, the CO adsorption at the central Fe site of both the **S7** and **Fe10** models is stronger than in the **S1** model, but is weaker than in the **Fe39** model where CO does not interact with Si atoms, suggesting that the CO adsorption in the *n*Fe/DASF model is greatly influenced by the surrounding Si atoms. The influence of Si atoms on CO adsorption is discussed in detail below.

In the **S7** case, the CO molecule binds to the central Fe site of the “7Fe wheel”, according to the **S7-CO-1** geometry, with a binding energy (*E_b_*_(CO)_) of −2.05 eV. The CO adsorption becomes weak when it binds with the Fe atom along the ring of the “7Fe wheel”, as is shown by the **S7-CO-2** geometry with an *E_b_*_(CO)_ value of −1.55 eV. Compared with **S7-CO-1** and **S7-CO-2**, the CO adsorption in **S7-CO-3** is much weaker. There is a C–Fe bond but no C–Si bond formed in **S7-CO-1** and **S7-CO-2**, whereas **S7-CO-3** has a C–Si bond but no C–Fe bond. In **S7-CO-3**, the C atom of CO binds to two Si atoms, where the C–Si distances are 1.917 Å (with the adatom Si) and 2.224 Å (with the other Si), very close to the result by Shong et al. [26]. In the cases from **S7-CO-1** to **S7-CO-3**, the C–O distance (*d*_C-O_) lengthens moderately from 1.178 Å to 1.183 Å and the C−O stretching frequency (*v*_CO_) decreases from 1924 cm^−1^ to 1811 cm^−1^. Therefore, the CO molecule prefers the Fe atom to the Si atom in the **S7** model.

When going from **S7-CO-1** to **S7-CO-2** and to **S7-CO-3**, the deformation energy of the CO (∆*E*_CO_) changes little; however, the deformation energy of the model (∆*E*_slab_) decreases greatly. The interaction energy (*E*_int_) between the model and the CO molecule becomes less negative from −3.12 eV to −1.99 eV and to −0.63 eV, suggesting that the charge transfer between the model and the CO molecule is expected to be weaker in the order of **S7-CO-1** > **S7-CO-2** > **S7-CO-3**. The Bader charge (*Q*_CO_) of CO is negative and becomes more negative from −0.250 *e* in **S7-CO-1** to −0.344 *e* in **S7-CO-2** and to −0.777 *e* in **S7-CO-3**, which is because the central Fe atom compared to the other Fe atoms is much more negatively charged (as is discussed in Section 2.1) and its *d* orbitals thus are occupied by more electrons than the other Fe atoms, suppressing its ability to accept electrons but promoting its ability to donate electrons. The charge of −0.777 *e* for CO in **S7-CO-3** is mainly contributed by the charge transfer from the Si atom to the CO molecule.

The CO adsorption on the **Fe10** model is very similar to the **S7** model, whether from the aspect of the *E_b_*_(CO)_ term or from the aspects of the *Q*_CO_, *d*_C-O_, and *v*_CO_ terms. The result shows that the adsorption of CO is little influenced by the permeation of three additional Fe atoms into the interlay, which is reasonable because each of the Fe atoms shares only one Si atom with the central Fe atom of the “7Fe wheel” and no Fe–Fe bond is formed between them. The ∆*E*_CO_ values are very similar for both models but the **Fe10** model deforms less than the **S7** model, which is consistent with the more negative *E*_b_ave_ value in the **Fe10** model than in the **S7** model (−5.087 eV vs. −5.032 eV), as well as the distribution of the electron density in these two models.

## 3. Methods and Materials

Spin-polarized DFT calculations are carried out by using the plane wave based pseudo-potential code in VASP [27,28]. The projector augmented wave method is used to calculate the electron–ion interaction [29,30]. The Perdew–Burke–Ernzerhof formalism is adopted to calculate the electron exchange-correlation energy [31]. The Kohn–Sham one-electron states in a plane wave basis set expanded up to 400 eV are employed with electron smearing with σ = 0.2 eV. The geometry optimization is converged with energy difference lower than 10^−4^ eV and forces smaller than 0.05 eV/Å. The Bader charge is calculated using the program developed by the Henkelman group [32].

The lattice parameters are calculated using the Si bulk crystal structure and its reciprocal space is sampled with a 15 × 15 × 15 k-point grid generated automatically using the Monkhorst–Pack method. The optimized bulk Si–Si bond is 2.36 Å in length. The DASF substrate is simulated with a Si(111) slab with a super cell of p(7×7), which contains seven layers of Si in the Z direction and 298 Si atoms. The bottom of the DASF substrate is passivated with one layer of 49 hydrogen atoms after optimizing the Si–H bonds, because hydrogen-termination of Si dangling bonds is one of the most common and useful methods of producing a chemically passivated surface [33,34,35,36,37]. During the optimization of the positions of Fe atoms, two bottom layers of Si together with the hydrogen atoms are fixed to model the bulk lattice properties, while the other Si atoms and Fe atoms are relaxed. The vacuum layer between periodically repeated slabs is set to be 15 Å to avoid interactions between slabs. The Brillouin zone is sampled with the Gamma-point.

The averaged binding energy (*E*_b_ave_) is calculated according to the equations *E*_b_ave_ = (*E_n_*_Fe/DASF_ − *E*_DASF_ − *nE*_Fe_)/*n*. The term *E_n_*_Fe/DASF_ is the total energies of the optimized DASF substrate with the adsorbed *n* Fe atoms in their equilibrium geometry; *E*_DASF_ is the total energy of the optimized DASF substrate, and *E*_Fe_ is the total energy of the Fe atom in the gas phase. All these energies are obtained in zero-temperature, static ground-state calculations, meaning that the entropy effect and phononic contributions to the free energy are not considered. The averaged Bader charge *Q*_ave_ of the Fe atoms is calculated according to the equation *Q*_ave_ = *Q_n_*_Fe_/*n*, where *Q_n_*_Fe_ represents the total Bader charge of the *n* Fe atoms on DASF.

## 4. Conclusions

In this work, the spin-polarized DFT method was employed to investigate the initial structure *n*Fe/DASF of the iron–silicon interface formed by the deposition of Fe atoms on the reconstructed Si(111)-(7×7) surface (named DASF). The stability of Fe atoms on the model was evaluated by averaged binding energies of Fe atoms. Two types of Fe growth were considered. One was that all the Fe atoms are adsorbed on the surface; the other was that Fe atoms freely permeate from the surface to the interlayer. In both cases, Fe atoms prefer to bind with Si atoms first rather than the adsorbed Fe atoms, because the Fe-Si interaction is stronger than the Fe-Fe interaction. In the first case, when increasing the *n* value, the average binding energy (*E*_b_ave_) generally becomes first more negative and then less negative, and reaches the minimum at *n* = 7 taking on a wheel-like 7Fe geometry. In the second case, a minimum *E*_b_ave_ value is presented at *n* = 10, with the additional three Fe atoms just below the 7Fe wheel. The variation in *E*_b_ave_ ~ *n* is similar to that of the averaged Bader charge against the *n* value, indicating that the bonding interaction determines the trends for the most part. The presence of the 7Fe wheel in both cases is mainly attributed to the enhanced Fe-Si interaction in the 7Fe wheel over the other geometries with the Fe atoms more dispersed. Because the central Fe site in both models of **S7** and **Fe10** is richer in electron density compared to other sites, CO adsorption occurs at the central Fe site of the 7Fe wheel, which is greatly influenced by the surrounding Si atoms but is little influenced by the additional Fe atoms in the interlayer. This work reveals the stability of the iron–silicon interface structure in the early stages of Fe growth on DASF. Also, the important properties such as the charge distribution and CO adsorption presented here are valuable for understanding and predicting the reactivity of the iron–silicon interface.

## Data Availability

Not applicable.

## Supplementary Materials

The following supporting information can be downloaded at: https://www.mdpi.com/article/10.3390/molecules28207181/s1, Table S1: The total energy (*E_n_*_Fe/DASF_) and the averaged binding energy (*E*_b_ave_) for the “**S*n***” models with all the Fe atoms on the DASF surface; Table S2: The total energy (*E_n_*_Fe/DASF_) and the averaged binding energy (*E*_b_ave_) for the “**Fe*n***” models with Fe permeation on the DASF surface; Coordinate files.

## Sample Availability

Samples of the models are available from the authors.
