# Supplementary material for: Theoretical Exploration of Properties of Iron–Silicon Interface Constructed by Depositing Fe on Si(111)-(7×7)
Source: Molecules. 2023 Oct 19;28(20):7181. doi: 10.3390/molecules28207181 (PMC10609458; doi:10.3390/molecules28207181)
Supplement: Supplementary file 1 [file molecules-28-07181-s001.zip › molecules-2639645-supplementary.pdf]

Supplementary Information for

Theoretical Exploration of Properties of Iron–Silicon Interface Constructed by Depositing Fe on Si(111)-(7×7)

Jun-Qing Yin [1],*, Yan-Ping Zhang [2], Yong You [1], Zhen-Hua Wang [1], Jian-Qiang Zhao [1] and Qing Peng [3]

1 Institute of Advanced Study, Chengdu University, Chengdu 610106, China; youyong@cdu.edu.cn (Y.Y.); wangzhenhua@cdu.edu.cn (Z.-H.W.); zhaojianqiang@cdu.edu.cn (J.-Q.Z.)
2 School of Pharmacy, Chengdu University, Chengdu 610106, China; zhangyanping@cdu.edu.cn
3 State Key Laboratory of Nonlinear Mechanics, Institute of Mechanics, Chinese Academy of Sciences, Beijing 100190, China; pengqing@imech.ac.cn
* Correspondence: yinjunqing@cdu.edu.cn

Table S1. The total energy ($E_{n\mathrm{Fe/DASF}}$) and the averaged binding energy ($E_{\mathrm{b\_ave}}$) for the "**S*n***" models with all the Fe atoms on the DASF surface, where n is the number of Fe atoms.

Model "**S*n***"	$E_{n\mathrm{Fe/DASF}}$ (eV)	$E_{\mathrm{b\_ave}}$ (eV)
**S1**	−1736.387769	−4.228
**S2**	−1744.376335	−4.409
**S3**	−1752.730515	−4.591
**S4**	−1760.970227	−4.654
**S5**	−1769.236751	−4.697
**S6**	−1778.445066	−4.882
**S7**	−1787.772033	−5.032
**S8**	−1795.566334	−4.952
**S9**	−1803.727181	−4.931
**S10**	−1812.424595	−4.968
**S11**	−1820.143884	−4.909
**S12**	−1828.500031	−4.913
**S13**	−1836.407191	−4.882
**S14**	−1843.877881	−4.824
**S15**	−1851.878756	−4.810
**S16**	−1859.300691	−4.760
**S17**	−1866.934091	−4.729

Table S2. The total energy ($E_{n\mathrm{Fe/DASF}}$) and the averaged binding energy ($E_{\mathrm{b\_ave}}$) for the "**Fe*n***" models with Fe permeation on the DASF surface, where n is the number of Fe atoms.

Model "**Fe*n***"	$E_{n\mathrm{Fe/DASF}}$ (eV)	$E_{\mathrm{b\_ave}}$ (eV)
**Fe1**	−1736.919476	−4.760
**Fe2**	−1744.916874	−4.680
**Fe3**	−1753.349987	−4.798
**Fe4**	−1761.495353	−4.785
**Fe5**	−1769.542338	−4.758
**Fe6**	−1778.445066	−4.882
**Fe7**	−1787.772033	−5.032
**Fe8**	−1796.452239	−5.063
**Fe9**	−1805.041663	−5.077
**Fe10**	−1813.612284	−5.087
**Fe11**	−1821.572245	−5.039
**Fe12**	−1829.706616	−5.014
**Fe13**	−1838.012209	−5.006
**Fe14**	−1846.051198	−4.980
**Fe15**	−1854.437957	−4.980
**Fe16**	−1862.433833	−4.956
**Fe17**	−1870.187944	−4.921
**Fe18**	−1879.063258	−4.952
**Fe19**	−1887.699004	−4.967
**Fe25**	−1935.001270	−4.851
**Fe28**	−1960.836422	−4.890
**Fe31**	−1985.325913	−4.878
**Fe35**	−2016.117906	−4.812
**Fe39**	−2047.713526	−4.780
